# The Critical Role of Nonhuman Primates in Medical Research

**DOI:** 10.20411/pai.v2i3.186

**Published:** 2017-08-23

**Authors:** Henry Friedman, Nancy Ator, Nancy Haigwood, William Newsome, James S. Allan, Thaddeus G. Golos, Jeff H. Kordower, Robert E. Shade, Michael E. Goldberg, Matthew R. Bailey, Paul Bianchi

**Affiliations:** 1 Duke University, Durham, North Carolina; 2 Johns Hopkins, Baltimore, Maryland; 3 Oregon Health & Science University, Portland, Oregon; 4 Oregon National Primate Research Center, Portland, Oregon; 5 Stanford University School of Medicine, Stanford, California; 6 Massachusetts General Hospital, Harvard Medical School, Boston, Massachusetts; 7 Wisconsin National Primate Research Center, Madison, Wisconsin; 8 Rush Medical College, Chicago, Illinois; 9 Texas Biomedical Research Center, San Antonio, Texas; 10 Columbia University Medical Center, New York, New York; 11 Foundation for Biomedical Research, Washington, DC; 12 Rock Unlimited, New York, New York

**Keywords:** Nonhuman primates, HIV, cancer, brain function, ethics, research

## Abstract

The sponsors of this report endorse carefully regulated research with nonhuman primates. This research is essential to learning about the biology, treatment and prevention of diseases and conditions that cause human suffering.

**Figure:**
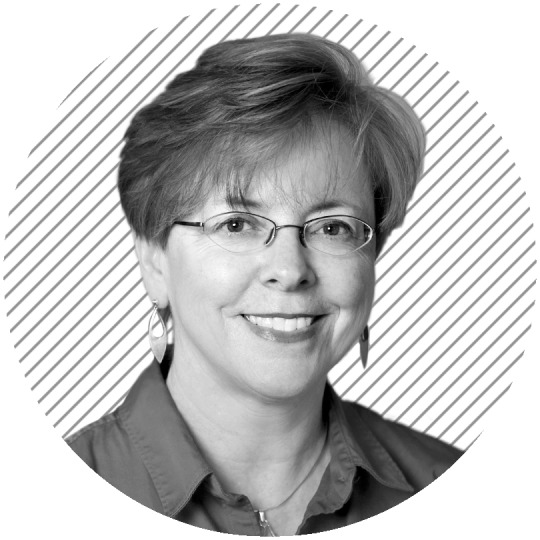

Research scientists are like detectives solving mysteries. We want to know why. More importantly, we want to save lives.

NANCY HAIGWOOD, PHD, DIRECTOR OF THE OREGON NATIONAL PRIMATE RESEARCH CENTER

Research with nonhuman primates (NHPs) – monkeys for the most part – has led to critical health advances that have saved or improved millions of human lives. While **NHPs account for just one-half of one percent** of animals in current medical research, it is no exaggeration to say they are essential to our ability to find cures for **cancer, AIDS, Alzheimer's, Parkinson's, obesity/diabetes,** and dozens of other diseases that cause human suffering and death.

Research with monkeys is critical to increasing our knowledge of how the human brain works and its role in cognitive, motor, and mental illnesses such as Alzheimer's, Parkinson's, and **depression**. This research is also fundamental to understanding how to prevent and treat emerging infectious diseases like **Zika** and **Ebola**. NHP research is uncovering critical information about the most common and costly metabolic disorder in the U.S. – **type 2 diabetes** – as well as the obesity that leads to most cases.

Without NHP research, we lose our ability to learn better ways to prevent negative pregnancy outcomes, including **miscarriage, stillbirth,** and **premature birth**. This research is also helping scientists to uncover information that makes human **organ transplants** easier and more accessible, literally giving new life to those whose kidneys, hearts, and lungs are failing.

## Monkeys Are Critical to All Stages of Research

News headlines tout medical breakthroughs. *Breakthrough* sounds dramatic, and to someone hearing about how the virus that causes polio is being used to put an aggressive form of **brain cancer** into remission, it is indeed. But as the scientists involved in that cancer research—and research into every other area of medicine—will tell you, breakthroughs might be dramatic, but they are never sudden.

A well thought-out and structured process is behind virtually every medical breakthrough and the discovery process probably took decades or more. Every step in the process was essential to the next, from basic research to human clinical trials.

**Figure:**
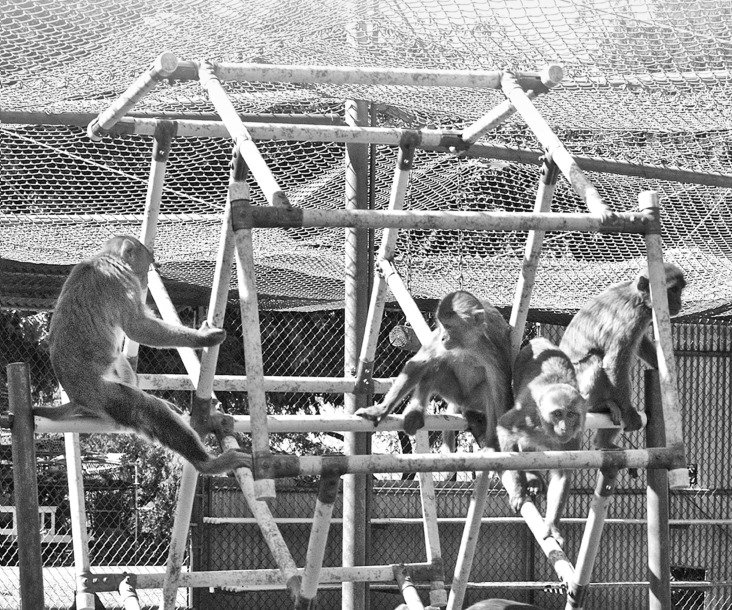
NONHUMAN PRIMATES USED IN MEDICAL RESEARCH The NHPs used in medical research are mainly macaques, a type of monkey that includes 23 species mostly found in Africa. Both are relatively small NHPs. Macaques generally weigh between 8 and 26 pounds with baboons slightly larger depending on the exact species. Great apes, such as chimpanzees, are no longer used in U.S. medical research.

PHOTO © KATHY WEST, CNPRC-UC DAVIS

Monkeys are often involved at the later stage of the process— what is called translational or applied research. Here all of the knowledge accumulated earlier is applied to specific medical questions such as: Will this vaccine protect a pregnant woman (and her baby) from Zika infection? And is the vaccine likely to be safe?

But monkeys also play a vital role in basic science research that can come decades earlier. Basic NHP research in the 1970s helped scientists understand the inner workings of the basal ganglia, the part of the brain that coordinates movement. Those early findings led to the “breakthrough” 30 years later in which deep brain stimulation is used to reduce involuntary movements of Parkinson's disease. See more breakthroughs linked to NHP research in Appendix A.

Regardless of where it occurs in the scientific discovery process, research with monkeys is highly regulated (see Appendix B). Scientists use monkeys only when no other research model can provide the required information. While rodents are used extensively and are extremely helpful in answering many basic research questions, their usefulness is limited by differences from primates in their lack of sophisticated brain structures, less developed immune systems and motor skills, and differences in how their metabolism functions, among other traits.

To cite an example, rodent brains are very different from human brains. The rodent lacks the prefrontal cortex specialization that is found in monkeys and humans. This difference limits the applicability of rodent studies in relation to studies of injury in the human brain.

Current studies in monkeys are helping to find ways to help **wounded soldiers** and **stroke** victims regain their independence after losing limbs or the ability to control them.

NHPs are also the main animals that allow quick response and research into emerging viruses, like Zika. What scientists learn about Zika itself, as well as what they learn about the best use of monkeys in Zika studies, they will apply to studies of future emergent diseases. And with recent history as a guide (Zika, Ebola, **Middle East Respiratory Syndrome [MERS], SARS, pandemic flu**, etc.), we should expect more infectious disease outbreaks in the near future.

## Focus on the Future: NHP Research Brings Hope to Millions of Patients

### Boosting the Body's Natural Defenses to Kill Cancer

**Figure:**
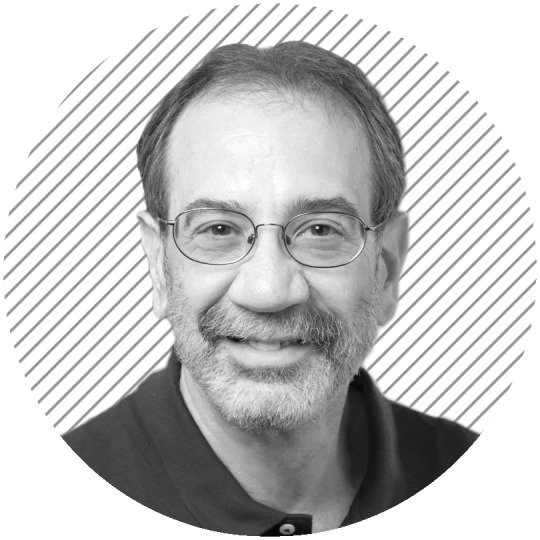

This is the most promising therapy I've seen in my career. Period.

HENRY S. FRIEDMAN, MD, INTERNATIONALLY RECOGNIZED NEURO-ONCOLOGIST, SPEAKING ABOUT USING MODIFIED POLIO VIRUS TO CURE BRAIN CANCER.

Modified poliovirus is being tested as a way to help the body's immune system see and destroy **glioblastomas**, the deadliest type of brain cancer. Glioblastomas can double in size every two weeks and can be deadly within months of diagnosis. The new treatment has led to complete remission in two glioblastoma patients and investigations are ongoing.

This type of research, called immunotherapy, uses the body's natural defense – the immune system – to destroy cancer cells. The harmless form of poliovirus is injected into glioblastoma tumors where it attaches to the cancer cells. The immune system recognizes the poliovirus as a dangerous invader and attacks – killing it and the cancer along with it.

It was 18 years from the earliest research until the first study in humans. Monkey research was essential in mapping out how to get the poliovirus through the brain and inside the cancerous tumors. The National Institutes of Health and the Food and Drug Administration also mandated testing the treatment first in monkeys to be sure the modified poliovirus would be harmless to humans.

**Figure:**
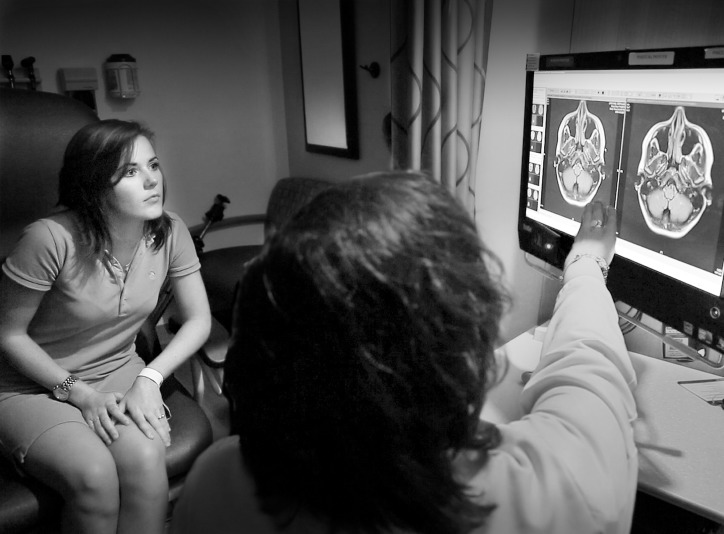
Several years ago, this young woman was diagnosed with a deadly brain tumor the size of a tennis ball. When conventional treatment didn't work her doctors suggested a new treatment developed with primate research. She agreed. Today, she's cancer free. PHOTO BY SHAWN ROCCO, FOR DUKE HEALTH NEWS AND COMMUNICATIONS

Doctors now are designing research to use this approach in treating many forms of cancer – including **breast** and **prostate cancers** – since the same receptor on the glioblastoma tumors that allows the poliovirus to attach itself also is found on virtually every cancerous tumor in humans.

### HIV/AIDS: Looking for a Vaccine and a Cure

**Figure:**
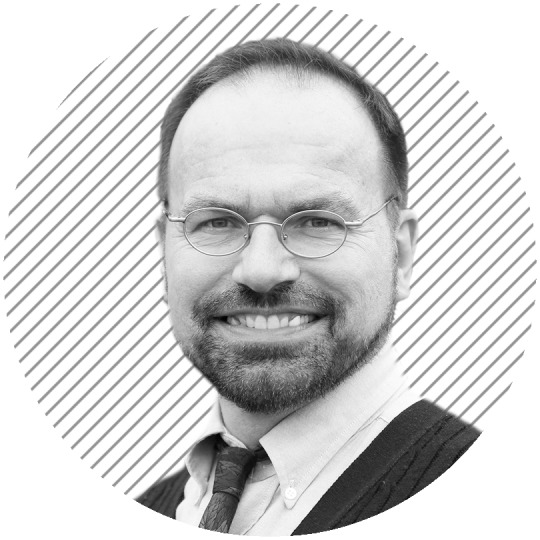

The value of studying monkeys is scientifically proven. HIV is no longer an impending death sentence.

KOEN VAN ROMPAY, DVM, PHD IS WORKING ON VACCINES AND ANTIVIRAL DRUGS TO TREAT OR PREVENT HIV INFECTION AND PEDIATRIC AIDS.

Scientists are looking for vaccines that can prevent **HIV** infection and treatments leading to a cure. Just 20 years ago medical advances changed the disease from a death sentence into a chronic, manageable disease. Drugs that keep the virus in check now give millions of HIV-infected people hope for a long and productive life.

Drug therapies effectively prevent HIV disease, but scientific advances in HIV are still needed. People with well-treated HIV still face more health problems than those without HIV [1, 2]. They age faster, too. Doctors estimate people with HIV are at least 5 to 14 years older than their chronological age [3, 4].

Monkeys are crucial to ongoing HIV research because of the combination of their unique biology among animals and their longevity, which is key in HIV studies that take from months to years to complete. Their similar biology helps scientists understand HIV disease, infection routes, the potential for vaccine-induced protection, and even an HIV cure.

An experiment reported in early 2016 looked at preventing **mother-to-child HIV transmission** [5]. After being exposed to simian-human immunodeficiency virus (SHIV), which is similar to HIV, infant monkeys with early stage infection were treated with human antibodies to block the infection. All of the monkeys in this experiment had no detectable virus in their blood or any of their tissues at the end of six months of observation.

In another experiment reported this year, rhesus macaques infected with another HIV-like virus were treated with standard anti-HIV medications plus an experimental drug that stimulates the immune system [6]. At the end of the study, 90 days after both medications were stopped, two monkeys showed no detectable virus in their bloodstream. This immune system stimulator was tested earlier in NHPs infected with chronic **hepatitis B**, leading to current research in humans with this potentially deadly infection. [7]

These studies hold promise for protecting babies from HIV infection and for finding a cure for those already infected, but much more research with monkeys will be needed to get there.

### Improving Pregnancy Outcomes

**Figure:**
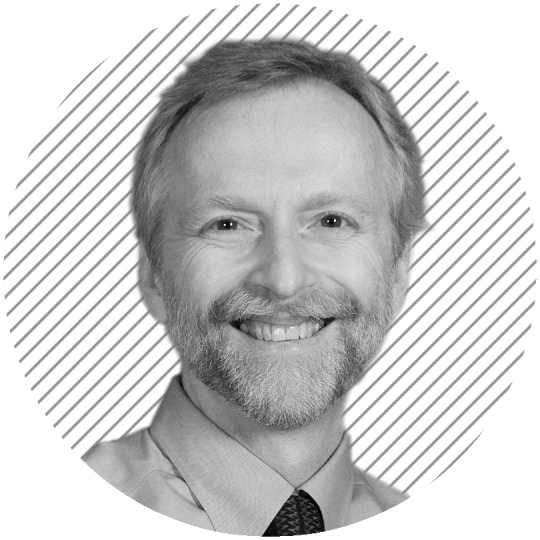

Primate research will help us learn new ways to prevent miscarriage, stillbirth, and premature babies.

THADDEUS GOLOS, PHD, COMPARATIVE BIOSCIENCES, OBSTETRICS AND GYNECOLOGY PROFESSOR AT UNIVERSITY OF WISCONSIN WHO IS WORKING TO UNDERSTAND THE MECHANISMS LEADING TO NEGATIVE PREGNANCY OUTCOMES.

In human clinical studies, a fundamental question is, “Do the potential benefits of this treatment outweigh the potential risks?” This question takes on added meaning when the study is in pregnant women. Researchers must not only consider the risks and benefits to the pregnant woman, but also to her developing fetus and ultimately to the child [8].

But how do researchers even begin to define these risks and benefits before human clinical trials? The answer is research with monkeys, since their fetal and placental development is uniquely similar to humans.

Researchers are working with macaque monkeys to understand the impact of Zika, the latest virus to emerge as a global threat. Zika infection in pregnant women can cause **microcephaly**, a condition where the child is born with a small head due to abnormal brain development. It also appears to cause stillbirth, miscarriages, and **fetal growth restriction**. These problems all appear to be rooted in how the Zika virus affects the developing fetus and the placenta, which nourishes the baby in its mother's womb.

The Zika virus infects monkeys just as it does humans, and both experience the disease in the same way. Researchers can study pregnant monkeys much as an obstetrician follows a woman's pregnancy – they can take blood, monitor fetal development through ultrasounds, and collect amniotic fluid. They can then test vaccines and drugs with the hope of protecting the fetus. No other animal model allows for this entire spectrum of study and application of the findings to pregnant women.

### Transplant Tolerance: The Next Big Step in Organ Transplant Success

More than 120,000 people in the U.S. are waiting for organ transplants and 22 of them die every day [9]. It is all the more tragic, then, when an organ transplant fails. This failure, or rejection, is caused when the recipient's immune system sees the new organ as “foreign” and attacks it.

To reduce the chance of organ rejection, transplant patients receive drugs to suppress their immune system. But the drugs come with a risk of toxicity and increase the risk of other problems, including development of cancers and infections resulting from a weakened immune system.

Research with monkeys is focused on achieving **transplant tolerance**— where the body's immune system does not see the new organ as foreign, thus eliminating the need for immunosuppressive drugs. While scientists have already made great strides in kidney transplant tolerance, they understand that tolerance is organ specific, so knowledge about the kidney may not transfer to the heart, lungs, liver, pancreas/pancreatic islets, or other types of transplants.

Transplant tolerance also differs by species. In other words, what works in a mouse may not work in a pig, and what works in a pig may not work in a monkey. Scientists learned about kidney transplant tolerance by starting with mice and then working up through swine and eventually into monkeys and humans. The same process is underway now for many other types of transplants.

### Mapping Out Brain Function

**Figure:**
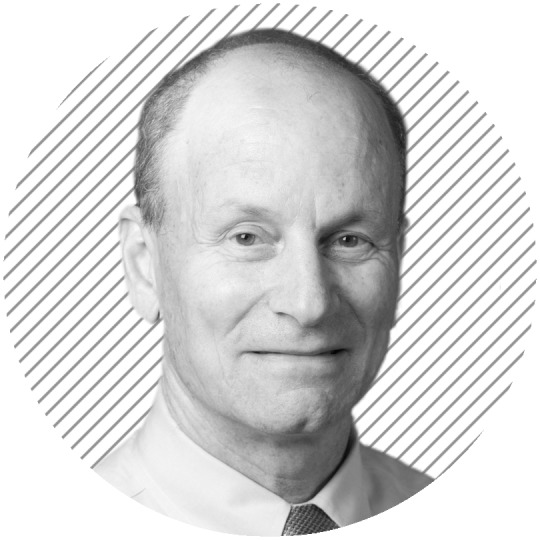

Understanding all brain diseases relies on us understanding how the healthy brain works during normal functioning.

STEPHEN LISBERGER, PHD, NEUROBIOLOGIST STUDYING HOW OUR BRAINS LEARN MOTOR SKILLS AND HOW WE USE WHAT WE SEE TO GUIDE HOW ME MOVE.

How does the brain work? No question could be more important for understanding human behavior and mental health, and for acquiring new information about the triggers in the brain that cause **psychiatric, movement** and **other neurological diseases**. The U.S. National Institutes of Health-supported BRAIN Initiative has developed a plan for improving our knowledge in these areas and research with monkeys and other species is critical to its success [10].

Scientists are mapping the activity of the billions of neurons deep inside the brain – the special cells that transmit the signals that drive thinking, mood, movement, and much more. By tracking neuron activity in monkeys while they are performing new tasks, scientists can actually see what parts of the brain are involved in sending the signals that take in, process, and store the newly acquired information.

What is unique to – or at least greatly enhanced by – the use of monkeys in this research is the range of cognitive behaviors that can be studied, the amount and precision of the data that can be collected, and the relevance of that data to human behavior and mental activity.

Seeing what is happening in a healthy monkey brain helps scientists understand what has gone wrong when a human brain is no longer working as it should. This type of research has relevance to Parkinson's disease and other movement disorders, all forms of **dementia**, including Alzheimer's, and behavioral and psychiatric problems from **alcoholism** and **attention-deficit disorder** to **bipolar disorder** and **autism**.

Alzheimer's and other dementias cost the U.S. $236 billion each year [11].

### Turning Science Fiction into Science Fact: Brain-Machine Interfaces

You see someone walking haltingly, dragging one leg behind him, or sitting with one arm draped listlessly on a table and immediately know he has had a stroke. Scientists learned long ago that it's not the muscles that are at fault; it's the nerve impulses inside the brain that have been affected.

A combination of scientific breakthroughs in neuroscience, computer processing and robotics has led to development of “brain-machine interfaces” – devices that allow humans to interact with their environment with prosthetic arms when they have lost the use of their own. Brain-machine interfaces translate signals in the brain into directions to move prosthetic arms.

Brain-machine interfaces can help paralyzed veterans interact with their environment.

This area of research shows enormous promise for humans who are paralyzed, such as injured veterans, or those with **brain damage** and **paralysis** due to stroke. As NHPs and humans have similarly developed brains and movements, experiments in monkeys have been vital to moving this field forward both conceptually and technically.

### Developing Vaccines for Babies and Adults

NHPs are essential to vaccine research. Among research animals, they alone can reproduce the entire biological process of the infections being studied. They allow researchers to monitor for information that is vital in understanding human infectious diseases – such as how a virus or bacterium reproduces inside the body, what symptoms it causes, and how the body's immune system responds to attack the invader.

Among the viruses currently in vaccine research trials is **respiratory syncytial virus**, or RSV – the most common cause of lower respiratory tract infections in U.S. infants and small children [12]. There is no treatment for RSV, [13] which hospitalizes nearly 60,000 U.S. children under age 5 every year and sends 2.1 million more to the doctor [14]. Vaccine research with monkeys is evaluating the safety of potential RSV vaccines in infants.

Other viruses under study include Ebola and **Marburg**, which can cause extreme bleeding that leads to death; the mosquito-borne **Dengue** and Zika viruses, capable of causing massive epidemics; and MERS plus the dangerous H5 and H7 **bird flu** strains, all of which have very high death rates.

### Baboons and Humans: Unique Connections for Blood Pressure Control

**Figure:**
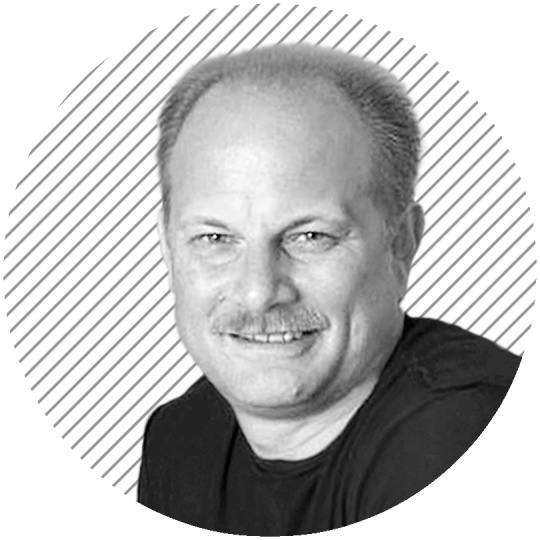

Monkeys have certain traits and characteristics that make them essential and irreplaceable in medical research. **They're the “bridge to the clinic.”**

JEFF KORDOWER, PHD, NEUROSCIENTIST EXAMINING HOW DISEASES LIKE PARKINSON'S AND ALZHEIMER'S AFFECT THE BRAIN TO CAUSE THEIR SYMPTOMS.

Lowering blood pressure is vitally important to individuals and our society. **High blood pressure** is a major factor in **heart disease** – the number one killer in the U.S. and the world [15]. And it's not just heart disease; high blood pressure leads to stroke, **kidney damage, memory problems**, and many other illnesses [16].

Decades ago, researchers made a breakthrough discovery that long-term blood pressure regulation is nearly identical in humans, baboons, and other NHPs. In fact, adult NHPs frequently develop hypertension similar to humans. Subsequent studies with monkeys have helped billions around the world lower blood pressure and reduce their risk of deadly complications.

Scientists recently discovered that baboons share another unique trait with humans – a characteristic in their red blood cells that can lead to salt-sensitivity and an inherited form of hypertension that is particularly difficult to treat. Current research is looking for new targets to control this type of high blood pressure.

Research with monkeys provides another key benefit – lifespan. High blood pressure becomes more common as we age and researchers are able to work with older baboons to gain essential information about the mechanisms driving this age-based increase – vital to the health of our aging population.

### Diabetes and Obesity: Connected in NHPs Just as in Humans

**Figure:**
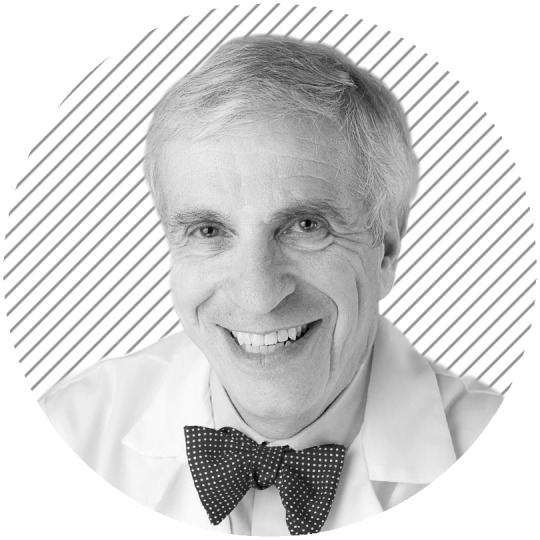

The top 3 benefits of our human/monkey research partnerships? Safety. Efficacy. And greater predictability.

MICHAEL GOLDBERG, MD, PROFESSOR OF BRAIN AND BEHAVIOR DEPARTMENTS AT COLUMBIA UNIVERSITY COLLEGE OF PHYSICIANS AND SURGEONS, WHO STUDIES HOW THE BRAIN PROCESSES WHAT THE EYES SEE.

Type 2 diabetes develops in monkeys just as it does in humans, even following the same age patterns, that is to say, more disease as we get older (one-fourth of U.S. seniors have diabetes) [17]. NHPs with diabetes even develop the same complications that are common in humans: eye disease, kidney disease, nerve damage and pain, and blood vessel disease, among others [18].

NHPs and humans have very similar systems that regulate blood sugar. For example, the structure and function of the group of cells in the monkey pancreas (called islets) that produce insulin are very similar to human islets. The islets in mice, rats, pigs, and other animals share some similarities with humans, but there are important differences, making monkeys a critical model for developing treatment and prevention methods, and for testing new therapies for people with diabetes.

Nonhuman primates are the ideal model for testing new therapies for people with diabetes, including the artificial pancreas, drugs and devices.

Type 2 diabetes and the U.S. obesity epidemic are linked – obesity is a contributing factor to the condition. More than a third of U.S. adults are obese and another third are overweight [19]. As with diabetes research, monkeys provide a critically important study model for human obesity. Monkeys that are fed a diet similar to the typical American diet respond like humans, gaining weight and later progressing to type 2 diabetes.

Researchers are examining the role of gastrointestinal proteins called glucagon-like peptides in the development of obesity in bonnet macaques. Bonnet macaques are unique among NHPs because they have a strong genetic predisposition to obesity. This research is looking for obesity treatments that will be as effective as invasive bariatric surgery, but with far less risk.

In conclusion, because NHPs are the most readily available models with the greatest psychological and genetic similarities to humans, they play an indispensable role in the process of medical research and development.

## Figures and Tables

**Figure:**
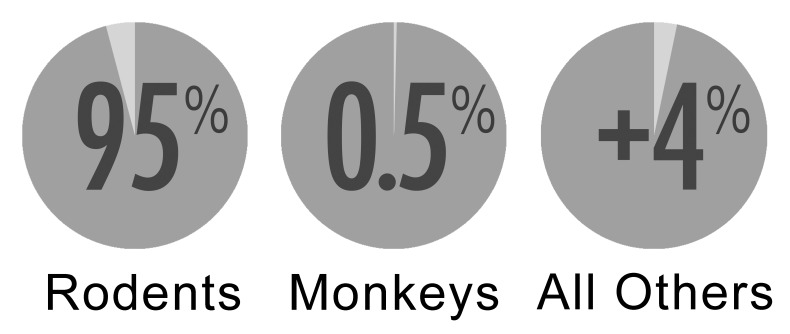
Lab Animals by Species The number of nonhuman primates used in research is less than 1%. But its impact on human health is enormous.

## Appendix A: Partial List of Scientific Advances Linked to Research in Nonhuman Primates

### 1900-1950s

- Components of blood and plasma discovered.
- Ability to diagnose and treat typhoid fever.
- Modern anesthesia.
- Mumps virus discovered.
- Treatment of rheumatoid arthritis.
- Discovery of the Rh factor, blood-typing knowledge critical for safe blood transfusions.
- Development of polio vaccine.
- Development of antipsychotic medication chlorpromazine and its tranquilizing derivatives.
- Cancer chemotherapy.
- Development of yellow fever vaccine.

### 1960s

- Mapping of the heart's connections to arteries.
- Development of German measles vaccine.
- Therapeutic use of cortisone for reducing inflammation and allergy symptoms.
- Corneal transplants.
- Development of treatment and prevention of radiation sickness.
- Development of measles, mumps, and rubella (MMR) vaccine.
- Discovery of the biochemical cause of depression.
- Transmissibility of human prion diseases, such as Creutzfeldt-Jacob disease, discovered.

All of our ability to address diseases in applied research comes from efforts in basic science research. WILLIAM NEWSOME, PHD, NEUROBIOLOGIST STUDYING HOW OUR BRAINS TRANSLATE WHAT WE SEE INTO MOVEMENT.

### 1970s

- Treatment of leprosy.
- Procedures to restore blood supply in the brain.
- Interaction between tumor viruses and genetic material.
- Understanding of slow viruses, which linger in the nervous system.
- Understanding of the inner workings of the basal ganglia, the part of the brain that coordinates movement.
- Discovery of mechanisms of opiate withdrawal and the anti-withdrawal effects of clonidine.
- Development of cyclosporine and other anti-rejection drugs helpful for organ transplants.

### 1980s

- Processing of visual information by the brain.
- Identification of physiological and psychological co-factors in depression, anxiety and phobias.
- Treatment of malnutrition caused by food aversion following chemotherapy.
- Treatment of congenital cataracts and “lazy eye” in children.
- First animal model for research on Parkinson's disease, enabling doctors to more accurately research human Parkinson's disease.
- Heart and lung transplant to treat cardiopulmonary hypertension.
- First hepatitis B vaccine.
- Development of rhesus monkey model for HIV/AIDS.
- Addition of taurine to infant formulas. Taurine is necessary for normal eye development.
- First treatment of naturally diabetic NHPs with a hormone-like insulin stimulus that is now in wide use both for diabetes and obesity treatment (GLP-1 agonist).

Without continued nonhuman primate research, many important translational discoveries in the field of transplantation will never happen. These preclinical investigations are critical to the many patients who are currently waiting for transplantation and the many others who are facing the possibility of organ rejection after transplantation. JAMES S. ALLAN, MD, THORACIC SURGEON AND CO-DIRECTOR OF THE CARDIOTHORACIC TRANSPLANTATION LABORATORY AT MASSACHUSETTS GENERAL HOSPITAL.

### 1990s

- Estrogen discovered to control an enzyme key to making serotonin, the brain chemical that regulates mood. Represents first step to providing effective medications for depression at the end of the menstrual cycle, and postpartum and postmenopausal depression.
- Demonstration of the effectiveness of early administrationof AZT to prevent or treat HIV infection. Thanks to this, HIV-infected mothers can give birth to HIV-free babies.
- Demonstration in monkeys of the high efficacy of the HIV drug tenofovir to prevent or treat infection.
- Lead toxicity studies help U.S. fight childhood lead exposure.
- Ongoing development of a one-dose transplant drug to prevent organ rejection.
- First controlled study to reveal that even moderate levels of alcohol are dangerous in pregnancy.
- Breakthroughs in understanding the mechanisms of puberty and disorders of puberty.
- Primate embryonic stem cells studied extensively for the first time, advancing efforts to better understand reproduction and genetic disorders.
- Control of intimal hyperplasia, a complication of coronary bypass surgery.
- Parent to child lung transplants for cystic fibrosis.
- NHPs shown to naturally develop diabetes, which is the same disease as in humans, thus opening the path to research for new treatments.
- Naturally regenerative mechanism discovered in the mature NHP brain, spurring new research toward curing Alzheimer's and other degenerative brain disorders.
- Development of anthrax vaccine.
- Development of life-saving medications for lupus.

### 2000s

- Gene that boosts dopamine production and strengthens brain cells used to successfully treat monkeys showing symptoms of Parkinson's disease.
- Monkey model developed to study the effects of malaria in pregnant women and their off-spring.
- NHPs are prime model for development of HIV treatments and potential vaccines.
- Insulin-treated diabetic patients live longer, fuller lives.
- The most common and debilitating complications of diabetes can now be studied in NHPs.
- High blood pressure is treated to prevent heart attack, stroke, and kidney failure.
- Patients can receive hip replacements and are no longer reliant on wheelchairs.
- People with degenerative eye diseases are able to see more clearly.
- Better medications improve lives of people with severe depression, bipolar disorder, and other psychiatric illnesses.
- Better pre- and postnatal care protects children.
- Earlier diagnoses and better treatments help those with polycystic ovary syndrome, endome triosis, and breast cancer.
- Improved treatments help more men survive prostate cancer.
- Secondhand smoke shown to affect prenatal, neonatal and child lung development, cognitive function and brain development.
- Exposure to wildfire smoke adversely affects development of the immune system.
- Better understanding of the effects of BPA, a chemical found in plastic, on prenatal development improves health of children and adults.

Adapted with permission from: Primate Info Net, National Primate Research Center, University of Wisconsin – Madison. At http://pin.primate.wisc.edu/research/discoveries.html.

## Appendix B: Regulating the Use of Nonhuman Primates in Research

Two federal agencies oversee how animals are used in medical research.

For **research funded by any Public Health Service entity**, such as the National Institutes of Health (NIH) or the National Science Foundation, the NIH:

→ Establishes *Public Health Service Policy on Humane Care and Use of Laboratory Animals*. [20]
→ **Mandates use of** the *Guide for the Care and Use of Laboratory Animals*, which is issued by the **National Academy of Science Institute for Laboratory Animal Research**. This guide addresses day-to-day aspects of caring for laboratory animals.[21]
→ Mandates that every institution appoints an Institutional Animal Care and Use Committee (see below).

The **USDA's Animal Plant and Health Inspection Service** enforces the *Animal Welfare Act* with unannounced compliance inspections of all regulated entities using animals in research, testing or teaching at least yearly [22].

The **Association for the Assessment and Accreditation of Laboratory Animal Care International** is an independent non-government accrediting organization. While voluntary, this accreditation includes broader requirements than the regulations. This demonstrates research facilities want to go “above and beyond” in the care of animals [23].

Today's researchers monitor not just the nutritional and environmental needs of NHPs but psychological needs, too. ROBERT SHADE, PHD, SCIENTIST EMERITUS AT SOUTHWEST NATIONAL PRIMATE RESEARCH CENTER

Every institution involved in nonhuman primate research is required by the *Animal Welfare Act* as well as Public Health Service policy to appoint and empower an **Institutional Animal Care and Use Committee**[24] that reviews and approves the research. Scientists must justify their use of primates and also explain why alternative forms of research (for example, studying cells or using computer simulations) are not able to achieve their scientific goals. They must also confirm that their research does not unnecessarily duplicate previous research [25].
